# Mathematical models of apparent viscosity as a function of water–cement/binder ratio and superplasticizer in cement pastes

**DOI:** 10.1038/s41598-023-48748-4

**Published:** 2023-12-15

**Authors:** Yong Yuan, Xiaoyun Wang, Xi Chen, Peng Xiao, Eduardus Koenders, Ying Dai

**Affiliations:** 1 College of Civil Engineering, Tongji University, Shanghai, 200092 China; 2 College of Civil Engineering and Architecture, Jiaxing University, Zhejiang, 314001 China; 3 Institute of Construction and Building Materials, Technical University of Darmstadt, 64287 Darmstadt, Germany; 4 School of Aerospace Engineering and Applied Mechanics, Tongji University, Zhangwu Road 100, Shanghai, 200092 China

**Keywords:** Civil engineering, Composites, Mechanical properties

## Abstract

The water–cement/binder ratio and the admixture of water-reducing agents strongly affect the rheological properties of cement pastes. This study develops mathematical models to predict the apparent viscosity of cement pastes with varying water-cement/binder ratios and polycarboxylate-based superplasticizer content by introducing the power law shear stress-shear strain relation of non-Newtonian fluids into the Navier–Stokes motion equations. The developed models are compared with the results of rheological experiments and verified for their accuracy in simulating the apparent viscosity of cement pastes. These models provide insight into the rheological behaviour of cement pastes and could have practical applications in the construction industry.

## Introduction

The rheology of fresh cement paste is closely linked to its workability, primarily determined by the water-cement ratio *(w/c)*^1–4^. After adding mineral admixtures to cementitious materials, the water-binder ratio *(w/b)* is used instead^5^. Numerous rheological models have been developed to characterize the rheological properties of cement pastes. For example, Einstein^6^ proposed a rigid sphere suspension model with a linear relationship between viscosity and solid volume fraction, closely related to *w/c* or *w/b*^7^. Other models with non-linear relationships, such as the power law (Krieger-Dougherty model), exponential law (Mooney model), and fraction forms (Eilers model, Robinson model, Quemada model, et al.)^8–10^, have been applied to describe non-linear results in cement pastes.

Several rheological models have been used to analyse the evolution of shear stress with shear rate and *w/c* in cement pastes or similar materials^4,11–17^. These models include the Bingham model which presents a linear relationship between shear stress and shear rate^11,13^, and the modified Bingham model which applies quadratic functions to discuss the non-linear parts of the shear stress-shear rate in fresh pastes^12^. Other models, such as the Casson model which adds a square root function of the shear rate to the Bingham model^13^, and the Ostwald model which uses a power law to obtain the whole constitutive relation between shear stress and shear rate^14^, have also been employed. The Herschel–Bulkey model combines the Bingham model and the Ostwald model^15,16^, while the Bingham–Papanastasiou model has a Papanastasiou’s function multiplier based on the Bingham model^4^. The Vipulanandan model takes a fraction function as the shear rate^17^. The parameters in these models are often adjusted based on experimental results from rheological tests with various shear protocols^14^, and they often change as *w/c* varies^4^.

**Figure 1:**
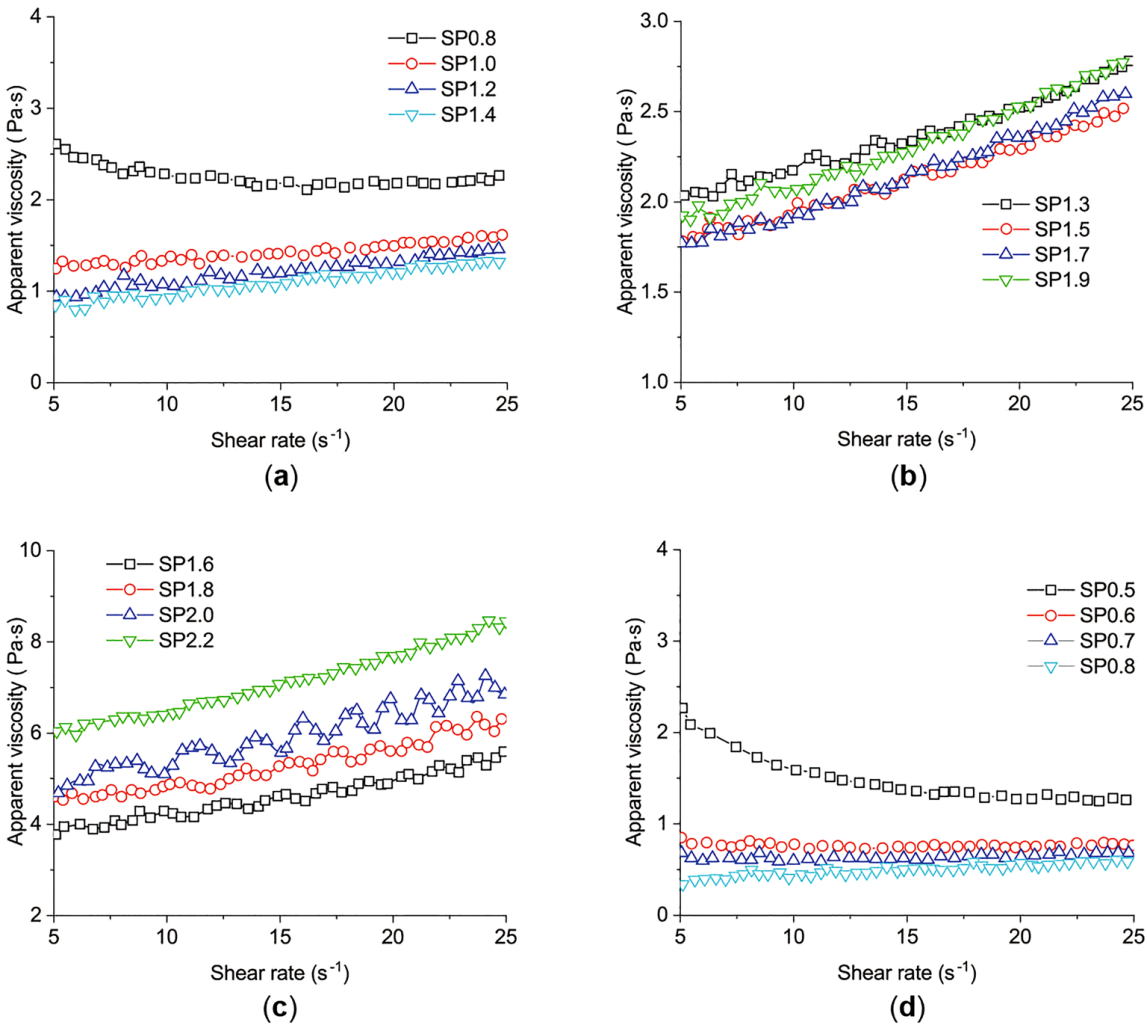
The relations between the apparent viscosity and the shear rate for pastes with different *w/b*^18^. (**a**) paste with w/b = 0.24, (**b**) paste with w/b = 0.20, (**c**) paste with w/b = 0.16, (**d**) paste with w/b = 0.32.

Apparent viscosity, an important rheological index, is influenced by several factors, including hydration degree, thixotropy, and the dosage of additives and admixtures^14^. The experimental rheological results of Liu et al.^18^, shown in Fig. 1, demonstrate the relations between the apparent viscosity and the shear rate of cement pastes. These relations change with the *w/b* and the dosage of superplasticizer (SP). The cement pastes can show shear thickening behavior^18^, for cement paste with lower *w/b* and a high dosage of SP, as shown in Fig. 1b and c.

Most of the present models are developed based on the test results in rheological experiments to consider the influence of *w/c*, additives, and admixtures. The representatives of the models are Jones and Taylor’s model (six-parameter exponential form)^19^, Lapasin et al. model (linear function of *w/c*)^20^, Ivanov and Roshavelov model (twenty-parameter polynomial with *w/c*, SP, silica fume, tricalcium aluminate and sulfur trioxide)^21^, and Rosquo$\ddot{\text{e}}$t model (linear function of *w/c* and power law of the shear rate)^22^. Other empirical formulations consider the yield stress in the Bingham model^14^, the Herschel–Bulkey model^14^, the YODEL model^23,24^, and the Ma & Kawashima model^25^ as a fractional or exponential function of the cement volume fraction.

Different from the experimental or empirical method, the target of this paper is to give a theoretical explanation of the change in the viscosity of cement pastes considering *w/c(w/b)* and SP based on mathematical derivations. Firstly, the Ostwald model is introduced into the Navier–Stokes equations of solid mixing with liquid. Next, an ordinary differential equation is derived by the mean value theorem in a tiny domain. To solve the ordinary differential equation, the Bernstein polynomial approximation is applied in the whole domain to derive a concise mathematical model. The model is a four-parameter formulation of the shear stress-shear rate for cement pastes with one variable *w/c*. More importantly, by introducing the electrostatic repulsion and steric hindrance into the Navier–Stokes equations, the influence of the SP can be quantitively considered by a function of its dosage. Correspondingly, the mathematical model can be extended to present the shear stress changes with the shear rate, *w/c*, and SP, with the same four-parameter formulation. The developed model is verified by being compared with the experimental results from Rosquo$\ddot{\text{e}}$t et al.^22^, Jeong et al.^4^, Cyr et al.^26^, and Liu et al.^18^.

## Method

### The apparent viscosity model with *w/c*

According to Kundu et al.^27^, the Navier–Stokes equations are

1 $$\frac{D\left(\rho {u}_{i}\right)}{Dt}=-\frac{\partial P}{\partial {x}_{i}}+\nabla \left(\eta \nabla {u}_{i}\right)+{\rho f}_{i}$$

in which $\frac{D}{Dt}$ is the material derivative, $\rho$ is the density of the fluid, $P$ is the liquid pressure, $\eta$ is the apparent viscosity of the fluid, ${u}_{i}$ is the velocity in the *x*_i_ (*i* = 1, 2, 3) direction, ${f}_{i}$ is the body force on a unit volume of the fluid. In the Cartesian coordinate system, *O-x*_*1*_*x*_*2*_*x*_*3*_, *i* (*i* = 1, 2, 3) corresponds to the *x*, y, and z axis respectively.

A simply unidirectional flow with constant pressure is considered, therefore the Newton’s law of viscosity is applicable as

2 $$\left\{\begin{array}{c}{u}_{1}=u\left(y, t\right)\\ {u}_{2}=0\\ {u}_{3}=0\\ P={P}_{0}\end{array}\right.$$

where ${u}_{1}$ depends on the space variable *y* and the time variable *t*, ${P}_{0}$ is the initial pressure. By substituting Eq. (2) into Eq. (1), Eq. (3) is obtained

3 $$\frac{\partial \left(\rho u\right)}{\partial t}=\frac{\partial }{\partial y}\left(\eta \frac{\partial u}{\partial y}\right)+{\rho f}_{x}.$$

Considering the procedure of mixing the solid and the liquid to produce fresh cement paste^28^, Eq. (3) is changed into

4 $$\frac{\partial \left({\phi }^{c}\rho u\right)}{\partial t}={\phi }^{c}\frac{\partial }{\partial y}\left(\eta \frac{\partial u}{\partial y}\right)+{\phi }^{c}\rho {f}_{x}$$

in which ${\phi }^{c}$, the volume fraction of liquid, can be taken as water in cement paste

5 $${\phi }^{c}=1-\phi$$

here $\phi$, the volume fraction of solid, can be taken as the binder particles for cement with additives such as silica fume and fly ash. The solid phase serves as the structural phase in the paste and is assumed to sustain the body force of the liquid phase. By introducing the drag force, we have

6 $$\frac{d}{dt}\left({\phi m}_{s}{u}_{s}\right)=-{\phi }^{c}{\rho f}_{x}$$

where ${u}_{s}$ and ${m}_{s}$ are the velocity and the mass of the solid respectively. In unit volume, ${m}_{s}$ can be expressed as

7 $${m}_{s}=\phi {\rho }_{s}$$

${\rho }_{s}$ is the density of the solid. Substituting Eqs. (5)–(7) into Eq. (4), it is obtained

8 $$\frac{\partial \left[\left(1-\phi \right)\rho u+\phi {\rho }_{s}{u}_{s}\right]}{\partial t}=\left(1-\phi \right)\frac{\partial }{\partial y}\left(\eta \frac{\partial u}{\partial y}\right).$$

In a tiny time increment $\Delta t$, the variables $\phi$ and ${\rho }_{s}$ own little changes. Ignoring the variation of $\phi$ and ${\rho }_{s}$, Eq. (8) can be written as

9 $$\frac{1}{\phi {\rho }_{s}}\frac{\partial \left[\phi {\rho }_{s}\left(\frac{\left(1-\phi \right)\rho }{\phi {\rho }_{s}}u+{u}_{s}\right)\right]}{\partial t}=\frac{\left(1-\phi \right)}{\phi {\rho }_{s}}\frac{\partial }{\partial y}\left(\eta \frac{\partial u}{\partial y}\right)$$

where *w/b* is given as

10 $$w/b=\frac{\left(1-\phi \right)\rho }{\phi {\rho }_{s}}.$$

Then, Eq. (9) is changed into

11 $$\frac{1}{\phi {\rho }_{s}}\frac{\partial \left[\phi {\rho }_{s}\left(\left(w/b\right)u+{u}_{s}\right)\right]}{\partial t}=\frac{w/b}{\rho }\frac{\partial }{\partial y}\left(\eta \frac{\partial u}{\partial y}\right).$$

The shear rate $\dot{\gamma }$ is

12 $$\dot{\gamma }=\frac{\partial u}{\partial y}.$$

Substituting it into Eq. (11), we have

13 $$\frac{1}{\phi {\rho }_{s}}\frac{\partial \left[\phi {\rho }_{s}\left(\left(w/b\right)u+{u}_{s}\right)\right]}{\partial t}=\frac{w/b}{\rho }\frac{\partial }{\partial y}\left(\eta \dot{\gamma }\right).$$

In pure cement pastes, the value of *w/b* is

14 $$w/b = w/c$$

According to Ostwald^14^, the power law shear stress-shear rate relation is

15 $$\tau \text{=}K{\dot{\gamma }}^{n}$$

where *K* and *n* are the calculation parameters. Then the apparent viscosity can be expressed as

16 $$\eta \text{=}\frac{\tau }{\dot{\gamma }}=K{\dot{\gamma }}^{n-1}.$$

In fact, the apparent viscosity in Eq. (16) can be affected by many factors, e.g., *w/c*, cement components, mixing time, standing time before measurement, and the degree of hydration^17,18^. When the cement components, the mixing time, the standing time before measurement, and the degree of hydration are determined, the viscosity is a function of the *w/c*

17 $$\eta \text{=}\eta \left(\text{w/c}\right)$$

which means the parameters *K* and *n* might be the function of *w/c* as

18 $$\left\{\begin{array}{c}K\text{=}K\left(\text{w/c}\right)\\ n=n\left(\text{w/c}\right)\end{array}\right. .$$

Substituting Eq. (16) into Eq. (13), we have

19 $$\frac{1}{\phi {\rho }_{s}}\frac{\partial \left[\phi {\rho }_{s}\left(\left(w/c\right)u+{u}_{s}\right)\right]}{\partial t}=\frac{w/c}{\rho }\frac{\partial }{\partial y}\left(K{\dot{\gamma }}^{n}\right).$$

In a tiny space increment $\Delta y$, it can be approximated as

20 $$K\approx {\left(\frac{\partial {\dot{\gamma }}^{n}}{\partial y}\right)}^{-1}\frac{\rho }{\phi {\rho }_{s}}\frac{\partial \left[\phi {\rho }_{s}\left(u+\frac{{u}_{s}}{\left(w/c\right)}\right)\right]}{\partial t}.$$

According to Eq. (10), the solid volume fraction $\phi$ is

21 $$\phi =\frac{\rho }{\left(w/c\right){\rho }_{s}+\rho }.$$

Substituting Eq. (21) into Eq. (20), it is obtained

22 $$K\approx {\left(\frac{\partial {\dot{\gamma }}^{n}}{\partial y}\right)}^{-1}\frac{\left(w/c\right){\rho }_{s}+\rho }{{\rho }_{s}}\frac{\partial \left[\frac{\rho {\rho }_{s}}{\left(w/c\right){\rho }_{s}+\rho }\left(u+\frac{{u}_{s}}{\left(w/c\right)}\right)\right]}{\partial t}.$$

In the $\Delta y$, $\frac{\partial {\dot{\gamma }}^{n}}{\partial y}$ is approximately considered as a constant. It means K can be determined by *w/c* in a tiny domain $({w}_{0}/{c}_{0},w/c)$, where ${w}_{0}/{c}_{0}$ is an arbitrary known water cement ratio in the concrete mix proportion of interest and $w/c$ is close to ${w}_{0}/{c}_{0}$. Then, $K$ can be given by the Lagrange mean value theorem in the tiny domain

23 $$K=K{|}_{w/c={w}_{0}/{c}_{0}}+{K}_{1}\left[\left(w/c\right)-\left({w}_{0}/{c}_{0}\right)\right]$$

in which

24 $${K}_{1}=\frac{\partial K}{\partial \left(w/c\right)}{|}_{w/c=\Delta w}$$

and

25 $$\Delta w={w}_{0}/{c}_{0}+\theta \left[\left(w/c\right)-\left({w}_{0}/{c}_{0}\right)\right]$$

where $\theta \in \left(0, 1\right)$. Substituting Eq. (23) into Eq. (22), it is derived

26 $$K{|}_{w/c={w}_{0}/{c}_{0}}+{K}_{1}\left[\left(w/c\right)-\left({w}_{0}/{c}_{0}\right)\right]={\left(\frac{\partial {\dot{\gamma }}^{n}}{\partial y}\right)}^{-1}\frac{\left(w/c\right){\rho }_{s}+\rho }{{\rho }_{s}}\frac{\partial \left[\frac{\rho {\rho }_{s}}{\left(w/c\right){\rho }_{s}+\rho }\left(u+\frac{{u}_{s}}{\left(w/c\right)}\right)\right]}{\partial t}.$$

In the increment $\Delta t$, $w/c$ owns little changes, the right side of Eq. (26) can be approximated as.

27 $${\left(\frac{\partial {\dot{\gamma }}^{n}}{\partial y}\right)}^{-1}\frac{\left(w/c\right){\rho }_{s}+\rho }{{\rho }_{s}}\frac{\partial \left[\frac{\rho {\rho }_{s}}{\left(w/c\right){\rho }_{s}+\rho }\left(u+\frac{{u}_{s}}{\left(w/c\right)}\right)\right]}{\partial t}\approx {\left(\frac{\partial {\dot{\gamma }}^{n}}{\partial y}\right)}^{-1}\frac{\rho }{{\rho }_{s}}\frac{\partial u}{\partial t}+{\left(\frac{\partial {\dot{\gamma }}^{n}}{\partial y}\right)}^{-1}\frac{\rho }{{\left(w/c\right)\rho }_{s}}\frac{\partial {u}_{s}}{\partial t}.$$

Then Eq. (26) can be changed as

28 $$\left(w/c\right)K{|}_{w/c={w}_{0}/{c}_{0}}+\left(w/c\right)a{K}_{1}\approx \left(w/c\right){\left(\frac{\partial {\dot{\gamma }}^{n}}{\partial y}\right)}^{-1}\frac{\rho }{{\rho }_{s}}\frac{\partial u}{\partial t}+{\left(\frac{\partial {\dot{\gamma }}^{n}}{\partial y}\right)}^{-1}\frac{\rho }{{\rho }_{s}}\frac{\partial {u}_{s}}{\partial t}$$

where

29 $$a=\left(w/c\right)-\left({w}_{0}/{c}_{0}\right).$$

Equation (28) can be simplified as

30 $$\left(w/c\right)K{|}_{w/c={w}_{0}/{c}_{0}}+\left(w/c\right)a{K}_{1}\approx {C}_{K}$$

here $${C}_{K}$$ is

31 $${C}_{K}=\left(w/c\right){\left(\frac{\partial {\dot{\gamma }}^{n}}{\partial y}\right)}^{-1}\frac{\rho }{{\rho }_{s}}\frac{\partial u}{\partial t}+{\left(\frac{\partial {\dot{\gamma }}^{n}}{\partial y}\right)}^{-1}\frac{\rho }{{\rho }_{s}}\frac{\partial {u}_{s}}{\partial t}.$$

In the tiny neighborhood of the ${w}_{0}/{c}_{0}$, the following approximation is given

32 $$\left\{\begin{array}{c}\left(w/c\right)K{|}_{w/c={w}_{0}/{c}_{0}}\approx \left({w}_{0}/{c}_{0}\right)K\\ \left(w/c\right){\left(\frac{\partial {\dot{\gamma }}^{n}}{\partial y}\right)}^{-1}\frac{\rho }{{\rho }_{s}}\frac{\partial u}{\partial t}\approx \left({w}_{0}/{c}_{0}\right){\left(\frac{\partial {\dot{\gamma }}^{n}}{\partial y}\right)}^{-1}\frac{\rho }{{\rho }_{s}}\frac{\partial u}{\partial t}\end{array}\right. .$$

The values of $\frac{\partial {\dot{\gamma }}^{n}}{\partial y}$ and $\frac{\partial u}{\partial t}$ are the constants when the shear rate keeps constant. Substituting Eq. (32) into Eq. (30), we have.

33 $$\left({w}_{0}/{c}_{0}\right)K+\left(w/c\right)a{K}_{1}\approx {C}_{K}.$$

By dividing both sides of Eq. (33) by ${w}_{0}/{c}_{0}$, Eq. (34) is obtained as

34 $$K\approx \frac{{C}_{K}}{{w}_{0}/{c}_{0}}+\left(w/c\right)\frac{{a}_{1}}{{w}_{0}/{c}_{0}}\frac{\partial K}{\partial \left(w/c\right)}$$

where

35 $${a}_{1}=-a.$$

Solving the ordinary Eq. (34), we have

36 $$K\approx {CC}_{\mathrm{I}}{\left(w/c\right)}^{{CC}_{\mathrm{II}}}+b$$

in which ${CC}_{\mathrm{I}}$ is the calculation parameter, ${CC}_{\mathrm{II}}$ and *b* are

37 $${CC}_{\mathrm{II}}=\frac{{w}_{0}/{c}_{0}}{{w}_{0}/{c}_{0}-w/c}\quad b=\frac{{C}_{K}}{{w}_{0}/{c}_{0}},$$

In the whole domain, the solution (36) is considered as the basic function. The Bernstein polynomial approximation^29^ can be concisely constructed as

38 $$K\approx {D}_{1}{\left(w/c\right)}^{{D}_{2}}+{D}_{3}$$

where *D*_1_, *D*_2_ and *D*_3_ are the calculation parameters, and the detailed derivation of Eq. (38) is shown in Appendix I (see the Supplementary Information Appendix I for details). Substituting Eq. (38) into Eq. (19), it is derived

39 $$\frac{1}{\phi {\rho }_{s}}\frac{\partial \left[\phi {\rho }_{s}\left(\left(w/c\right)u+{u}_{s}\right)\right]}{\partial t}\approx \frac{w/c}{\rho }\frac{\partial }{\partial y}\left(\left({D}_{1}{\left(w/c\right)}^{{D}_{2}}+{D}_{3}\right){\dot{\gamma }}^{n}\right).$$

Expanding Eq. (39), we have

40 $$\frac{1}{\phi {\rho }_{s}}\frac{\partial \left[\phi {\rho }_{s}\left(\left(w/c\right)u+{u}_{s}\right)\right]}{\partial t}\approx \frac{\left(w/c\right)\left({D}_{1}{\left(w/c\right)}^{{D}_{2}}+{D}_{3}\right)}{\rho }n{\dot{\gamma }}^{n-1}\frac{\partial \dot{\gamma }}{\partial y}.$$

Then, *n* can be expressed as

41 $$n\approx \frac{\rho }{\left(w/c\right)\left[{D}_{1}{\left(w/c\right)}^{{D}_{2}}+{D}_{3}\right]}{\left[\frac{1}{\phi {\rho }_{s}}\frac{\partial \left[\phi {\rho }_{s}\left(\left(w/c\right)u+{u}_{s}\right)\right]}{\partial t}\right]\left({\dot{\gamma }}^{n-1}\frac{\partial \dot{\gamma }}{\partial y}\right)}^{-1}$$

which is the implicit solution of *n*. The solution is complicated and therefore hard for engineering applications. For simplification, the *n* is expanded in the neighborhood of $w/c={w}_{0}/{c}_{0}$ as

42 $$n\approx {f}_{0}+{f}_{1}\left(w/c-{w}_{0}/{c}_{0}\right)+{f}_{2}{\left(w/c-{w}_{0}/{c}_{0}\right)}^{2}+\cdots$$

and the first-order approximation is

43 $$n\approx {f}_{0}+{f}_{1}\left(w/c-{w}_{0}/{c}_{0}\right)$$

in which ${f}_{0}$ is

44 $${f}_{0}=n{|}_{w/c={w}_{0}/{c}_{0}}$$

and $${f}_{1}$$ is

45 $${f}_{1}=\frac{\partial n}{\partial \left(w/c\right)}{|}_{w/c={w}_{0}/{c}_{0}}.$$

It is taken the parameters as

46 $${C}_{1}={f}_{1},\quad {C}_{2}={f}_{0}-\left({w}_{0}/{c}_{0}\right){f}_{1} .$$

When the value of ${f}_{0}$ is

47 $${f}_{0}=\left({w}_{0}/{c}_{0}\right){f}_{1}$$

which means the parameter ${C}_{2}=0$, and *n* is

48 $$n\approx {C}_{1}{w}_{0}/{c}_{0}.$$

Due to the arbitrariness of ${w}_{0}/{c}_{0}$, the general form of *n* is presented as.

49 $$n\approx {C}_{1}w/c.$$

Substituting Eq. (38) and Eq. (49) into Eq. (16), the four-parameter constitutive model is given as.

50 $$\eta =\left[{D}_{1}{\left(w/c\right)}^{{D}_{2}}+{D}_{3}\right]{\dot{\gamma }}^{{C}_{1}w/c-1}.$$

Equation (50) describes the functional relation between the shear stress and the shear rate of cement pastes with varying $w/c$. It should be noticed that the $w/c$ can be replaced with $w/b$ based on the assumption in Eq. (14).

### The apparent viscosity model with SP

**Figure 2:**
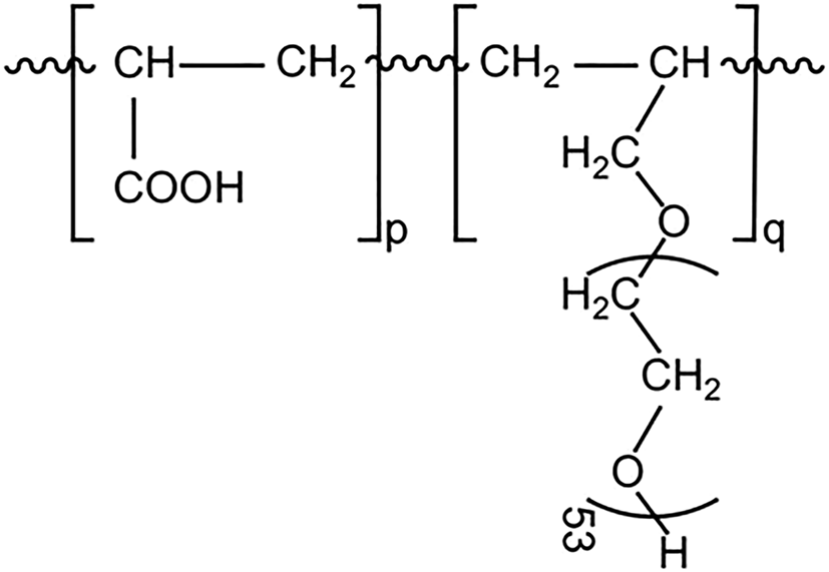
The chemical structure of the main component of the SP^18^.

This section discusses the apparent viscosity model with the SP which has the chemical structure as shown in Fig. 2. Its specific density is 1.07 g/cm^3^, with the side chain length (average number of ethylene oxide units) of 53. The average molecular weight is 58.2 × 10^3^ g/mol, and the polydispersity index is 2.0^18^.

The SP mainly disperses cement particles through electrostatic repulsion and steric hindrance after adsorption^30,31^. The electrostatic repulsion force ${F}_{e}$ is

51 $${F}_{e}=k\frac{{q}_{1}{q}_{2}}{{r}^{2}}$$

where $k$ is the Coulomb’s constant, ${q}_{1}$ and ${q}_{2}$ are the charges of ions, $r$ is the distance between two ions. When the type of SP is given, the $k$, ${q}_{1}$ and ${q}_{2}$ are known. The distance $r$ is related to the number or the concentration of ions in a certain space, which means the whole electrostatic repulsion force ${F}_{et}$ is

52 $${F}_{et}=\underset{\Omega }{\overset{}{\int }}k\frac{{q}_{1}{q}_{2}}{{r\left({n}_{S}\right)}^{2}}\mathrm{d}V$$

in which ${n}_{S}$ is the dosage of the SP in a fluid domain $\Omega$. The steric hindrance describes how the physical structure of SP affects its ability to react. Its force ${F}_{s}$ can be approximated as a constant for the SP. Introducing ${F}_{et}$ and ${F}_{s}$ into Eq. (4), it is obtained

53 $$\frac{\partial \left({\phi }^{c}\rho u\right)}{\partial t}={\phi }^{c}\frac{\partial }{\partial y}\left(\eta \frac{\partial u}{\partial y}\right)+{\phi }^{c}\rho {f}_{x}+{F}_{n}.$$

where $${F}_{n}$$ is.

54 $${F}_{n}={F}_{et}+{\phi }^{c}{F}_{s}.$$

According to Eq. (52), ${F}_{et}$ is related to the dosage of the SP, which means ${F}_{n}$ in Eq. (54) is the function of ${n}_{S}$. Substituting Eqs. (6)–(7) into Eq. (53), we have

55 $$\frac{\partial \left[\left(1-\phi \right)\rho u+\phi {\rho }_{s}{u}_{s}\right]}{\partial t}-{F}_{n}=\left(1-\phi \right)\frac{\partial }{\partial y}\left(\eta \frac{\partial u}{\partial y}\right)$$

In a tiny domain $\left(y,y+\Delta y\right)$, Eq. (55) can be approximated as

56 $$\frac{1}{\phi {\rho }_{s}}\frac{\partial \left[\phi {\rho }_{s}\left(\left(w/b\right)u+{u}_{s}\right)\right]}{\partial t}+\frac{1}{\phi {\rho }_{s}}{F}_{n}\approx \frac{w/b}{\rho }\frac{\partial }{\partial y}\left(\frac{\partial u}{\partial y}\right)\eta$$

which can be changed into

57 $$\eta \approx {\left[\frac{w/b}{\rho }\frac{\partial }{\partial y}\left(\frac{\partial u}{\partial y}\right)\right]}^{-1}\left[\frac{1}{\phi {\rho }_{s}}\frac{\partial \left[\phi {\rho }_{s}\left(\left(w/b\right)u+{u}_{s}\right)\right]}{\partial t}+\frac{1}{\phi {\rho }_{s}}{F}_{n}\right].$$

When the influence of the SP is ignored, ${F}_{n}$ equals zero. The apparent viscosity $\eta$ in Eq. (57) is reduced to Eq. (13), which can be approximated as the four-parameter model from Eq. (50).

58 $$\eta =\left[{D}_{1}{\left(w/c\right)}^{{D}_{2}}+{D}_{3}\right]{\dot{\gamma }}^{{C}_{1}w/c-1}.$$

Based on Eq. (57), it is considered that the influence of SP comes from the additional item ${\left[\frac{w/b}{\rho }\frac{\partial }{\partial y}\left(\frac{\partial u}{\partial y}\right)\right]}^{-1}\frac{1}{\phi {\rho }_{s}}{F}_{n}$, which also can affect the other parameters in Eq. (58). A modified item is added to illustrate the effect of SP in Eq. (58) and the expression of the apparent viscosity is constructed as

59 $$\eta =\left[{D}_{1}{\left(w/c\right)}^{{D}_{2}}+{D}_{3}\right]{\dot{\gamma }}^{{C}_{1}w/c-1}+{f}_{3}\left(w/b,{n}_{S}\right).$$

where ${f}_{3}\left(w/b,{n}_{S}\right)$ is the additional modified item, in $\left(y,y+\Delta y\right)$, ${f}_{3}$ is

60 $${f}_{3}\left(w/b,{n}_{S}\right)={\left[\frac{w/b}{\rho }\frac{\partial }{\partial y}\left(\frac{\partial u}{\partial y}\right)\right]}^{-1}\frac{1}{\phi {\rho }_{s}}{F}_{n}.$$

According to the Bernstein first-order approximation (see the Appendix I for details), considering the effect of SP to ${D}_{1}$ and ${C}_{1}$, the viscosity of whole domain in an arbitrarily known *y* position is approximated as

61 $$\eta \approx {F}_{1}\left({n}_{S}\right)\left[{d}_{1}{\left(w/b\right)}^{{D}_{2}}+{d}_{3}\right]{\dot{\gamma }}^{{F}_{2}\left(w/b,{n}_{S}\right)w/b-1}+{F}_{3}\left(w/b,{n}_{S}\right)$$

in which ${d}_{1}$ and ${d}_{3}$ are the calculation parameters. ${F}_{1}\left({n}_{S}\right)$ is the effective coefficient of the dosage of the SP, and ${F}_{3}\left(w/b,{n}_{S}\right)$ owns the same dimension of ${\left[\frac{w/b}{\rho }\frac{\partial }{\partial y}\left(\frac{\partial u}{\partial y}\right)\right]}^{-1}\frac{1}{\phi {\rho }_{s}}{F}_{n}$. The detailed expressions of ${F}_{1}$, ${F}_{2}$ and ${F}_{3}$ are shown in Appendix II (see the Supplementary Information Appendix II for details).

### The verification of the developed models with varying w/c (or w/b) and SP

In this section, the developed models are verified by being compared with the examples of the experimental results in rheological tests with different *w/c* and SP of cement pastes.

### Different *w/c* (or *w/b*) for cement pastes

Three examples are given from the experimental results of Rosquo$\ddot{\text{e}}$t et al.^22^, Cyr et al.^26^, and Jeong et al.^4^ to verify the developed model with *w/c*.

In the experiment of Rosquo$\ddot{\text{e}}$t et al.^22^, the Portland cement CEM I 52.5 PM ES CP2 is used. The Bogue compositions of the cement are listed as follows: ${\text{C}}_{3}{\text{S}}$ 63.30%, ${\mathrm{C}}_{2}\mathrm{S}$ 17.90%, ${\mathrm{C}}_{3}\mathrm{A}$ 4.74%, and ${\mathrm{C}}_{4}\mathrm{AF}$ 5.62%^22^.All measurements used a Rheomat 115 rheometer with an MS 145 coaxial cylinder spindle. A thermal control system maintains a constant temperature in the test. Tests were conducted at 0.5 min after mixing and at a temperature of 20 ± 1 $^\circ \mathrm{C}$. For each *w/c*, the measurements are conducted by increasing the shear rate from 23 s^−1^ to 1200 s^−1^. For each shear rate, the shear stress was measured after a minimum of 30 s without fluctuation^22^. Four experimental testing points with *w/c* = 0.5 were chosen as the collocation points to solve the calculation parameters in Eq. (50) as

62 $${\text{D}}_{1}\text{=-0.791497}, {\mathrm{D}}_{2}=1.001, {\mathrm{D}}_{3}=1.36931, {\mathrm{C}}_{1}=-0.88272.$$

**Figure 3:**
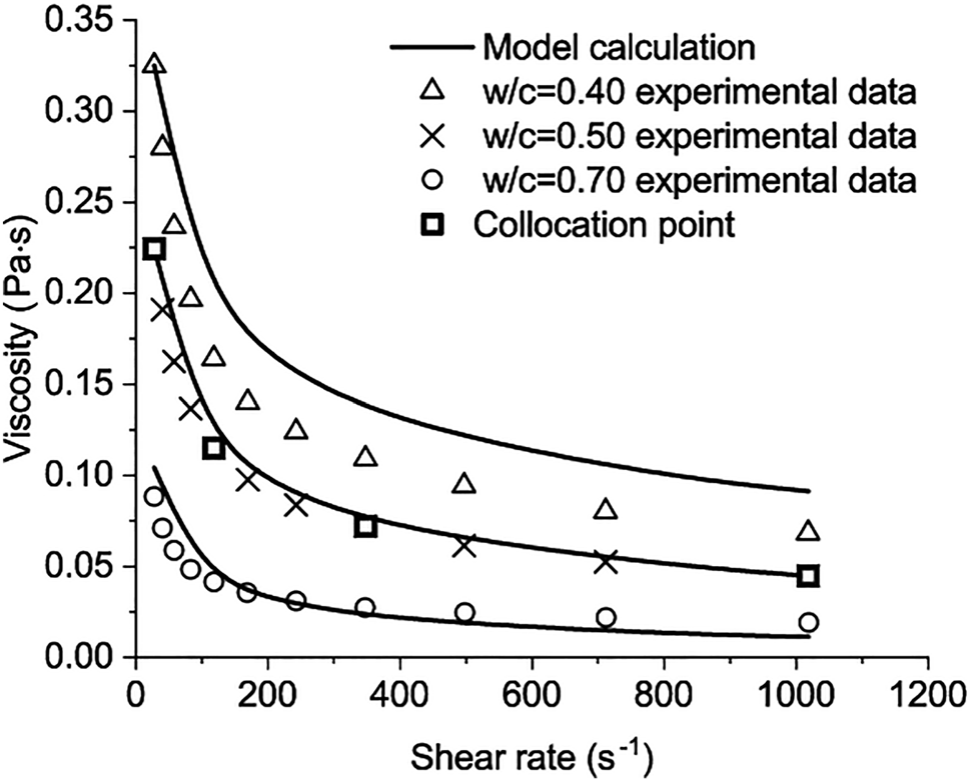
The comparison between model calculation and the experimental data when w/c = 0.4, 0.5, 0.7.

**Figure 4:**
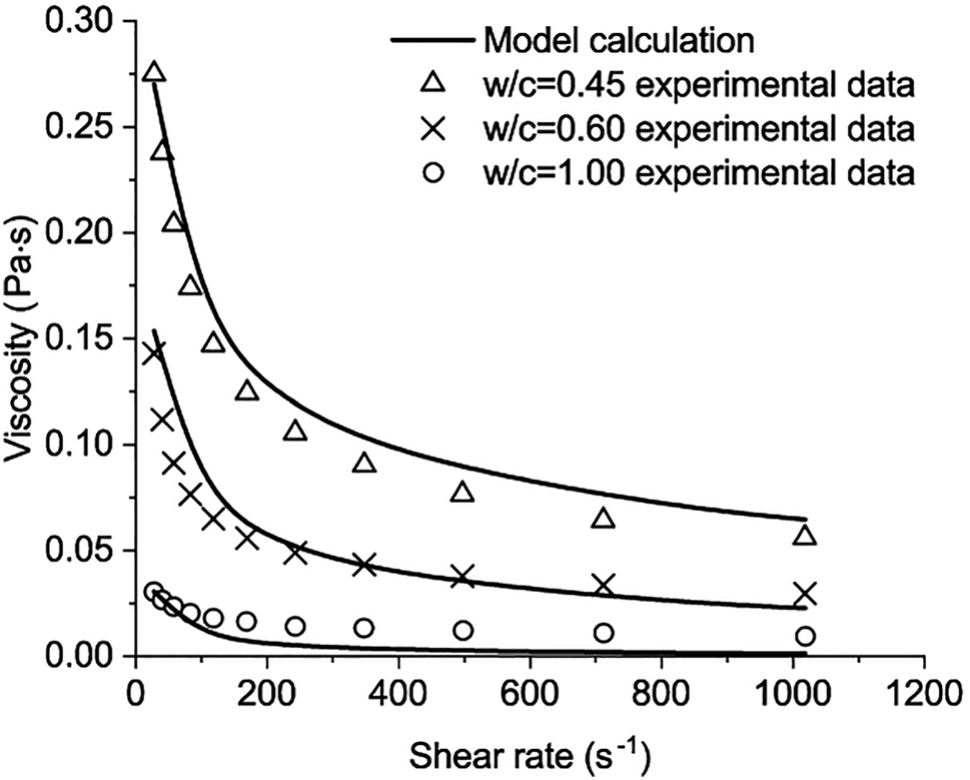
The comparison between model calculation and the experimental data when w/c = 0.45, 0.60, 1.00.

Compared with the other results in the experiment, the results of the developed model are shown in Figs. 3 and 4 In the two figures, the values of the apparent viscosity plunge before the shear rate reaches 100 s^−1^. Then, the descending rates slow when the shear rate further increases from 200 s^−1^ to 1000 s^−1^. The maximum viscosity is about 0.325 Pa*·s* for *w/c* = 0.4. The calculated results agree with the measured^22^.

The second verification is conducted based on the measured results from Cyr et al.^26^. The Bogue compositions of the cement used are listed as follows: ${\mathrm{C}}_{3}\mathrm{S}$ 60%, ${\mathrm{C}}_{2}\mathrm{S}$ 13%, ${\mathrm{C}}_{3}\mathrm{A}$ 10%, ${\mathrm{C}}_{4}\mathrm{AF}$ 6%, Gypsum 5%, and others 6%^26^. The apparatus used was a modified Rotovisco RV2 (Haake) with a six-blade vane. The pastes were mixed for 8.5 min to obtain a good dispersion of the components^26^. When the influence of gypsum and other ingredients is ignored, the value of *w/c* equals that of *w/b*. There are four experimental points of the curve with *w/b* = 0.3 used to solve the parameters in Eq. (50) as

63 $${\mathrm{D}}_{1}=0.154807, \quad {\mathrm{D}}_{2}=-5.3451966, \quad {\mathrm{D}}_{3}=-0.989956, \quad {\mathrm{C}}_{1}=1.174112.$$

**Figure 5:**
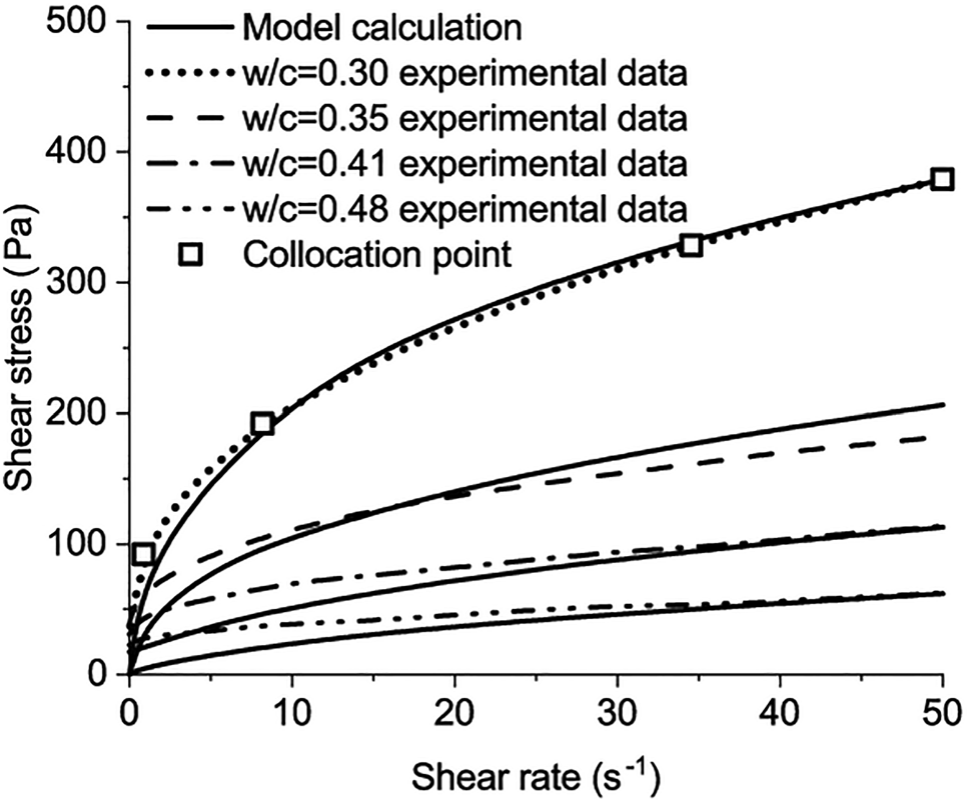
The comparison between model calculation and the experimental data when w/b = 0.30, .035, 0.41, 0.48.

The calculated results of the developed model and the measured results are shown in Fig. 5. The curve with *w/b* = 0.3 shows that the shear stress increases from 0 to 379 Pa with the shear rate increasing. The calculated results match the measured results^26^.

The third verification is based on the test results from the experiment of Jeong et al.^4^. The chemical compositions of the cement used are listed as follows: $\mathrm{CaO}$ 60.84%, ${\mathrm{SiO}}_{2}$ 13.26%, ${\mathrm{Na}}_{2}\mathrm{O}$ 10.05%, ${\mathrm{SO}}_{3}$ 3.59%, ${\mathrm{Al}}_{2}{\mathrm{O}}_{3}$ 3.42%, ${\mathrm{Fe}}_{2}{\mathrm{O}}_{3}$ 3.14%, $\mathrm{MgO}$ 2.35%, ${\mathrm{K}}_{2}\mathrm{O}$ 1.17%, and others 2.18% (all by the weight percentage of the cement)^4^. The matrix was mixed at low speed for 1 min, halting for 1.5 min, and then was mixed for another 0.5 min at high speed. The matrix was placed in the rheometer right after the mixing. The protocol of the rheological test consists of pre-shearing for 0.5 min at a shear rate of 500 s^−1^. The pre-shearing procedure avoids memory effects, e.g., the thixotropic effect^4^. Four experimental results of the shear stress-shear rate curve with *w/c* = 0.6 are chosen as the collocation points to solve the calculation parameters in Eq. (50) as

64 $${\mathrm{D}}_{1}=0.344347, \quad {\mathrm{D}}_{2}=-6.112651, \quad {\mathrm{D}}_{3}=0.75678866, \quad {\mathrm{C}}_{1}=0.77509.$$

**Figure 6:**
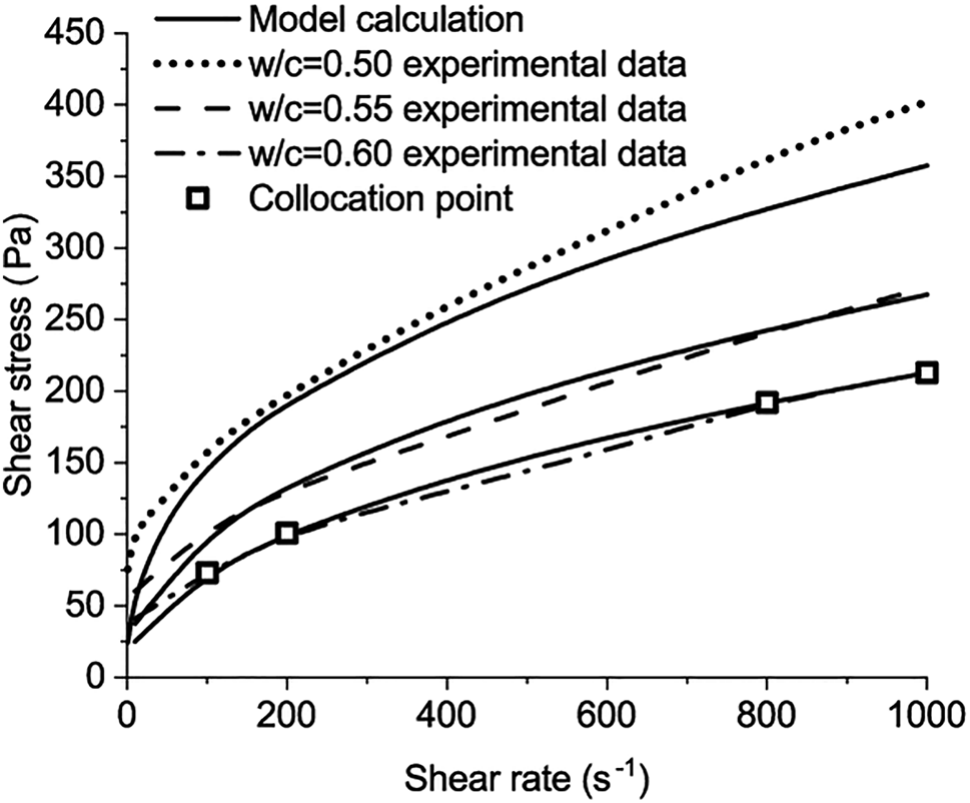
The comparison between model calculation and the experimental data when w/c = 0.50, 0.55, 0.60.

**Figure 7:**
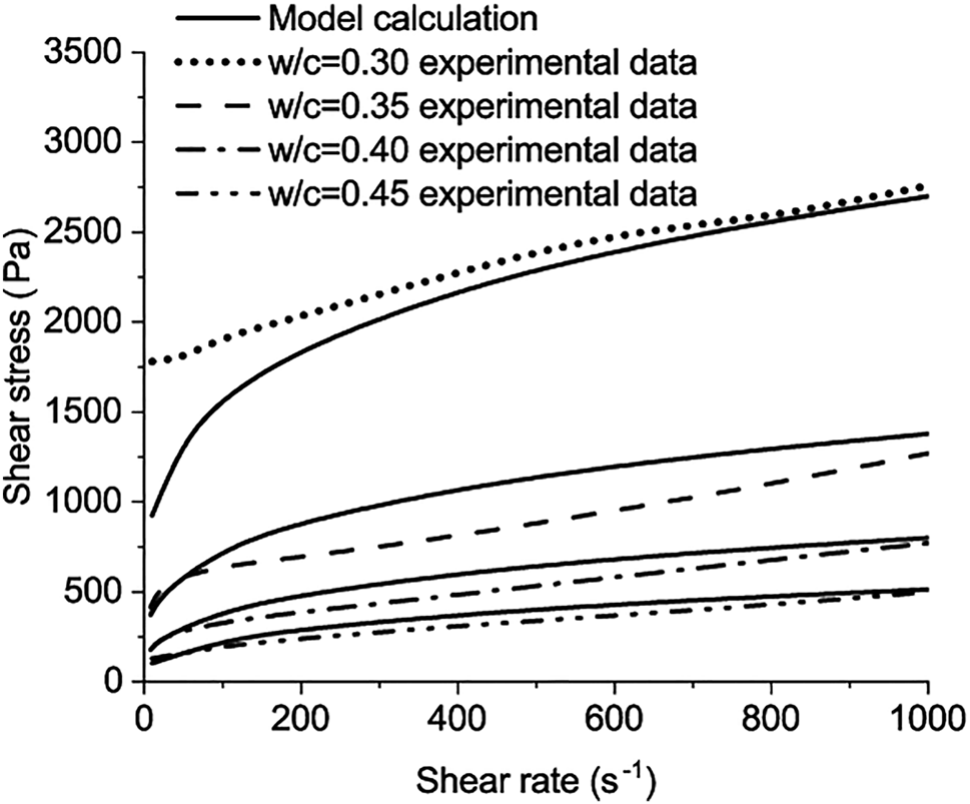
The comparison between model calculation and the experimental data when w/c = 0.3, 0.35, 0.40, 0.45.

Compared with the other measured results in the experiment of Jeong et al.^4^, the calculated results of the developed model are presented in Figs. 6 and 7.

The shear stress increases as the shear rate increases, which is applicable for pastes with *w/c* from 0.3 to 0.6, as shown in Figs. 6 and 7. The maximum shear stress is around 2600 Pa, measured in the paste with *w/c* = 0.3 at the shear rate of 1000 s^−1^. The difference can be observed between the calculated results of the developed model and the measured results at low shear rates, especially for the paste with *w/c* = 0.3. Two reasons result in the difference. First, the test results of cement paste with w/c = 0.6 were used to calibrate the four parameters of the model. Based on these parameters, the rheological behaviours of the other pastes were predicted and compared with the experimental results. For the cement paste with w/c = 0.3, its rheological behaviour is different from the paste with w/c = 0.6. When the w/c = 0.3, the paste behaves like a solid-like system before the shear stress reaches the static yield stress of the paste. After the shear stress exceeds the static yield stress, the paste starts to flow. For the paste with w/c = 0.6, the paste is nearly liquid-like and it starts flowing without a clear-cut yield stress^4^. The parameters are calibrated from the paste with w/c = 0.6 and lead to the variation for the paste with w/c = 0.3 at the low shear rate. As the shear rate increases, the flow resistance from the yield stress of the paste accounts for diminishingly and the variation reduces.

The second reason lies in the measurement protocol in Jeong’s research. In the measurement, fresh samples were left standing for 0.5 min after the preshear procedure. The 0.5 min is regarded as the minimum time interval to wait between the preshear and the successive measurement^4^. Then, the rotational speed of the rheometer starts to increase from zero. The paste changed from a flow state in the pre-shearing to a static state at the end of the standing time. The standing time enables the paste with w/c = 0.3 to recover to a solid-like system with static yield stress. Based on the abovementioned two points, the variation between test results and the proposed models can be observed for the paste with w/c = 0.3.

### Different *w/b* and SP for cement pastes

The test results from the experiment of Liu et al.^18^ in Fig. 1 were used for another verification. The chemical compositions of the cement used are listed as follows: $\mathrm{CaO}$ 63.80%, ${\mathrm{SiO}}_{2}$ 19.41%, ${\mathrm{Al}}_{2}{\mathrm{O}}_{3}$ 4.33%, ${\mathrm{SO}}_{3}$ 3.89%, ${\mathrm{Fe}}_{2}{\mathrm{O}}_{3}$ 2.91%, $\mathrm{MgO}$ 1.29%, ${\mathrm{Na}}_{2}\mathrm{O}$ 1.29%, ${\mathrm{K}}_{2}\mathrm{O}$ 0.68%, and ${\mathrm{TiO}}_{2}$ 0.28% (all by the weight percentage of the cement)^18^.

Four groups of mixed proportions are chosen with different *w/b* and SP, which are presented in Table 1.

**Table 1:** Mix proportions^18^.

Groups	Water-binder ratio	SP Dosage (%)	Cement (%)	Silica fume (%)	Ultra-fine slag (%)
Group 1	0.32	0.5, 0.6, 0.7, 0.8	75	10	15
Group 2	0.24	0.8, 1.0, 1.2, 1.4			
Group 3	0.20	1.3, 1.5, 1.7, 1.9			
Group 4	0.16	1.6, 1.8, 2.0, 2.2			

The apparent viscosity, defined as the ratio of shear stress to the shear rate, was measured using a Brookfield R/S SST2000 rheometer with Spindle CC25. Since the shear rate during concrete pouring was from 10 s^−1^ to 20 s^−1^, the maximum shear rate for the pastes was set to 25 s^−1^^18^. After placing the paste into the rheometer, the sample was left to equilibrate for 0.5 min and then sheared at a constant rate of 25 s^−1^ for 1 min. The collocation points are chosen from the curve with SP = 2 and *w/b* = 0.16, and the calculation parameters are solved as

65 $${\mathrm{d}}_{1}=-17.78758, \quad {\mathrm{D}}_{2}=0.78808, \quad {\mathrm{d}}_{3}=7.396649, \quad \uplambda =-4.301, \quad {\mathrm{\alpha }}_{1}=1.17071, \quad {\mathrm{\alpha }}_{2}=-11.72098, \quad {\mathrm{\alpha }}_{3}=136.3292, \quad {\upbeta }_{1}=0.11854, \quad {\upbeta }_{2}=-2.98819, \quad {\upbeta }_{3}=17.8869$$

**Figure 8:**
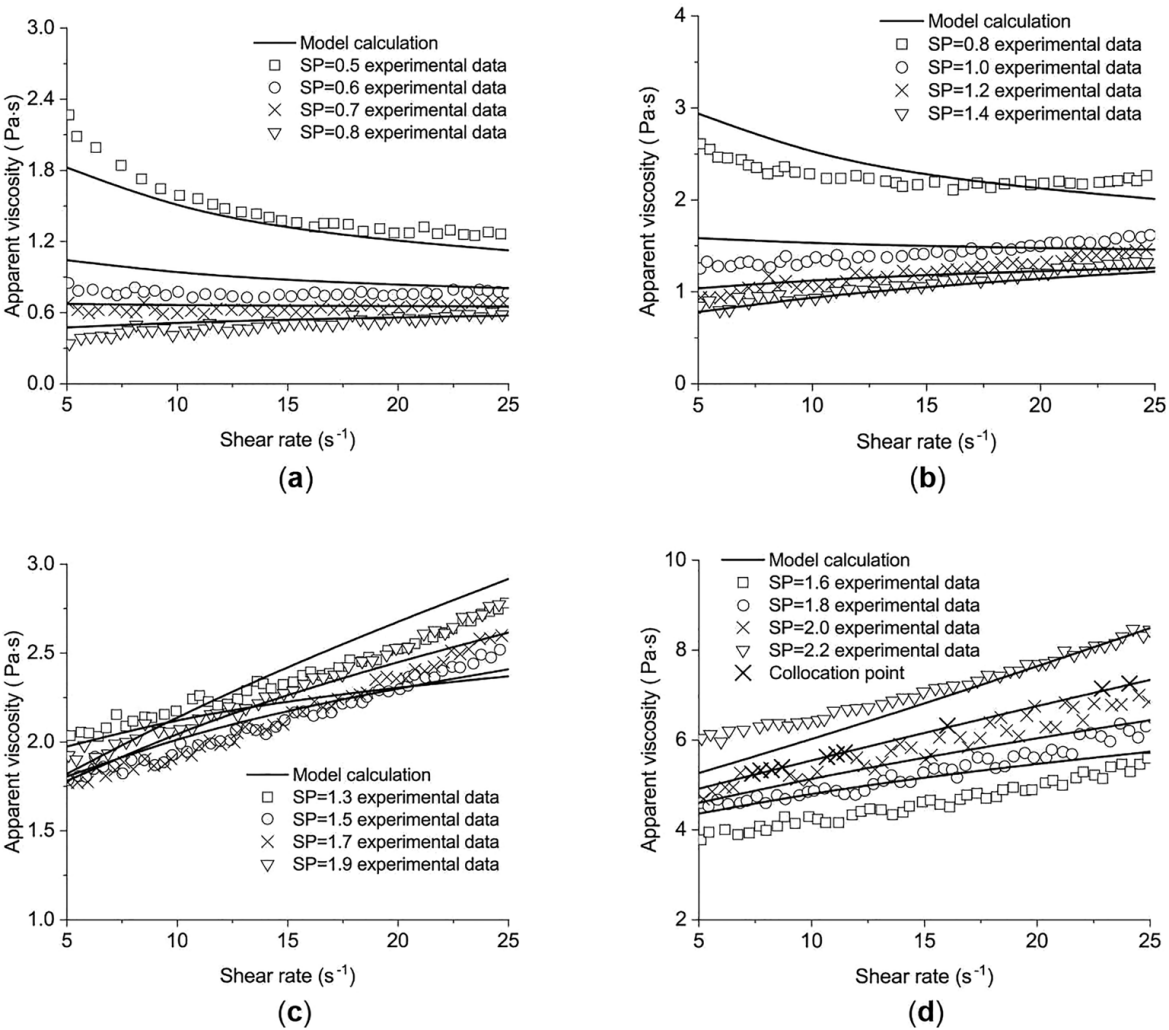
The comparison between model calculation and the experimental data for different w/b and SP. (**a**) paste with w/b = 0.32, (**b**) paste with w/b = 0.24, (**c**) paste with w/b = 0.20, (**d**) paste with w/b = 0.16.

Compared with the measured results, the calculated results of the present model are shown in Fig. 8. Figure 8 shows the changes in the viscosity of cement pastes with different *w/b* and SP. When *w/b* = 0.32, the viscosity monotonically decreases with SP = 0.5% (the minimum SP dosage). The other curves in Fig. 8a change from flat to monotonically increasing as the increase of the shear rate. The same phenomena are observed in Fig. 8b and c. In Fig. 8d, all curves monotonically increase as the shear rate increases. The maximum value of the viscosity is 8.448 Pa·s with *w/b* = 0.16, SP = 2.2%, and a shear rate of 25 s^−1^. The results of the developed model have the same trend of viscosity and agree with the experimental data.

The models can be used to calculate the viscosity of cement pastes and give technique support for the pumping process of cement pastes, e.g., long-distance pumping^32^, oil-well cementing^14^, and cementitious pastes grouting^33^. In these applications, the viscosity of the cementitious materials is important. With the models proposed in the paper, the effect of adjusting the water-cement ratio and adding superplasticizer on the viscosity of the pastes can be quantified and serves these applications.

## Conclusions

The paper presents a mathematical model for the apparent viscosity of cement pastes with varying water-cement/binder ratios and the influence of polycarboxylate-based superplasticizers (SP). The following conclusions can be drawn:

(1) An ordinary differential equation is developed considering the *w/b* and parameter K in the Ostwald model. It is derived based on the Navier–Stokes equations and the Ostwald model of shear stress-shear rate relations;

(2) The whole domain constitutive relation of a four-parameter formula of cement pastes in the rheological stage is approximated by the first-order Bernstein polynomial approximation with different water-cement/binder ratios;

(3) An approximate expression of the viscosity for one type of polycarboxylate-based superplasticizer is constructed considering the result of electrostatic repulsion and steric hindrance;

(4) The developed models are verified by several rheological experiments with different water-cement/binder ratios and dosages of superplasticizers.

It should be noted that the developed models are applicable during the initial mixing stage of cement pastes. Further studies are needed to consider the influence of other factors such as the degree of hydration and the addition of aggregates and other additives on the rheological behaviour of cement-based mixtures.

### Supplementary Information

Supplementary Information.

## Data Availability

The datasets used and/or analysed during the current study are available from the corresponding author upon reasonable request.

## Abbreviations

$$\frac{D}{Dt}$$: Material derivative
$\rho$: Density of fluid
$$P$$: Liquid pressure
$$\eta$$: Apparent viscosity
${u}_{i}$: Liquid velocity in *i* (*i* = 1, 2, 3) direction
${f}_{i}$: Body force on unit volume
*t*: Time variable
${P}_{0}$: Initial water pressure
$\phi$: Volume fraction of solid
${\phi }^{c}$: Volume fraction of water
*w/b*: Water–binder ratio
*w/c*: Water–cement ratio
${u}_{s}$: Velocity of solid
${m}_{s}$: Mass of solid
${\rho }_{s}$: Density of solid
$$\nabla$$: Laplace operator
$$\tau$$: Shear stress
$$\dot{\gamma }$$: Shear rate
*K*: Coefficient of Ostwald model
*n*: Index of Ostwald model
*k*: Coulomb’s constant
${q}_{1}$*,*${q}_{2}$: Charges of ions
*r*: Distance of two ions
${F}_{et}$: Electrostatic repulsion force
${n}_{S}$: Dosage of superplasticizer
${F}_{s}$: Steric hindrance force
