# Supplementary material for: Mathematical models of apparent viscosity as a function of water–cement/binder ratio and superplasticizer in cement pastes
Source: Sci Rep. 2023 Dec 15;13:22301. doi: 10.1038/s41598-023-48748-4 (PMC10724167; doi:10.1038/s41598-023-48748-4)
Supplement: Supplementary file 1 — Supplementary Information. [file 41598_2023_48748_MOESM1_ESM.docx]

**Appendix I: The Bernstein polynomials approximation for the whole domain**

In the whole domain, the Bernstein polynomials approximation of coefficient $K$ is

$K_{t0}\left( \xi\right)\approx\sum_{i=0}^{n} K_{t0}\left( \frac{i}{n} \right)\left[ \left( \begin{matrix} n \\ i \end{matrix} \right)\xi^{i}\left( 1-\xi\right)^{n-i} \right]$ (AI.1)

where $\left( \begin{matrix} n \\ i \end{matrix} \right)$ is binomial, $K_{t0}\left( \frac{i}{n} \right)$ and $\xi$ $\in\left[ 0,1 \right]$ are given as

$\left\{ \begin{aligned} K_{t0}\left( \frac{i}{n} \right)=\frac{K_{t}\left( \frac{i}{n} \right)}{K_{\mathrm{tmax}}} \\ \xi=\frac{\mathrm{CC}_{I}\left( w/c \right)^{\mathrm{CC}_{\mathrm{II}}}+b}{K_{\max}} \end{aligned} \right.$ (AI.2)

where $K_{t}$ is the coefficient $K$ in a sub domain, $K_{\mathrm{tmax}}$ and $K_{\max}$ are the maximum value of $K$ in the whole domain and the sub domain respectively. According to the characteristics of the Bernstein polynomials, it is always uniformly convergent to the true $K$ value in the whole domain. It is taken the first-order approximation of Equation (AI.1)

$K_{t0}\approx K_{t0}\left( 0 \right)+\left[ K_{t0}\left( 1 \right)-K_{t0}\left( 0 \right) \right]\xi$. (AI.3)

Then the approximation of coefficient $K$ in the whole domain is

$K\approx K_{t0}=D_{1}\left( w/c \right)^{D_{2}}+D_{3}$ (AI.4)

where

$\left\{ \begin{aligned} D_{1}=\frac{\mathrm{CC}_{I}}{K_{\max}}\left[ K_{t0}\left( 1 \right)-K_{t0}\left( 0 \right) \right] \\ D_{2}=\frac{{w_{0}}/{c_{0}}}{a_{1}} \\ D_{3}=\frac{C_{K}}{{w_{0}}/{c_{0}}}+K_{t0}\left( 0 \right) \end{aligned} \right.$. (AI.5)

In Equation (AI.5), $D_{i}$ (i=1, 2, 3) are expressed by $\mathrm{CC}_{I}$, $K_{\max}$, $K_{t0}\left( 1 \right)$, $K_{t0}\left( 0 \right)$, ${w_{0}}/{c_{0}}$, $a_{1}$ and $C_{K}$, which are the constants in solving. It does not need to be solved these constants separately, but can be obtained $D_{i}$ by collocation points from measurement.

**Appendix II: Constructing the approximation functions in the model with *w/b* and SP**

The $F_{1}\left( n_{S} \right)$ is expanded as

$F_{1}\left( n_{S} \right)=\sum_{i=0}^{+\infty} U_{i}\xi^{i}$ (AII.1)

where $\xi$ is

$\xi={n_{S}}^{\lambda}$ (AII.2)

$\lambda$ is the calculation parameter. According to Lapasin et al., the binary functions $F_{2}\left( w/b,n_{S} \right)$ and $F_{3}\left( w/b,n_{S} \right)$ are expanded as

$F_{2}\left( w/b,n_{S} \right)=\sum_{i=0}^{+\infty} h_{i}\left( w/b \right){n_{S}}^{i}$ (AII.3)

$F_{3}\left( w/b,n_{S} \right)=\sum_{i=0}^{+\infty} \varphi_{i}\left( w/b \right){n_{S}}^{i}$. (AII.4)

The first-order approximation is taken for simplification. The functions $F_{1}\left( n_{S} \right)$, $F_{2}\left( w/b,n_{S} \right)$ and $F_{3}\left( w/b,n_{S} \right)$ are

$F_{1}\left( n_{S} \right)\approx\left\{ \begin{aligned} {n_{S}}^{\lambda}, n_{S}>0 \\ 1, n_{S}=0 \end{aligned} \right.$ (AII.5)

$F_{2}\left( w/b,n_{S} \right)\approx\left\{ \begin{aligned} h_{1}\left( w/b \right)n_{S}, n_{S}>0 \\ C_{1}, n_{S}=0 \end{aligned} \right.$ (AII.6)

$F_{3}\left( w/b,n_{S} \right)\approx\varphi_{0}\left( w/b \right)+\varphi_{1}\left( w/b \right)n_{S}$. (AII.7)

Equation (59) can be reduced to Equation (58) when $n_{S}=0$. Substituting Equation (AII.7) into Equation (59) and set $n_{S}$=0, we have

$\varphi_{0}\left( w/b \right)=0$. (AII.8)

Then $h_{1}\left( w/b \right)$ and $\varphi_{1}\left( w/b \right)$ are constructed as

$\left\{ \begin{aligned} h_{1}\left( w/b \right)\approx\alpha_{1}\left( w/b \right)^{-1}+\alpha_{2}w/b+\alpha_{3}\left( w/b \right)^{3} \\ \varphi_{1}\left( w/b \right)\approx\beta_{1}\left( w/b \right)^{-2}+\beta_{2}+\beta_{3}\left( w/b \right)^{2} \end{aligned} \right.$ (AII.9)

in which $\alpha_{i}$ and $\beta_{i}$ (i=1,2,3,4) are the calculation parameters which can solved by collocation point method.
